# Anchoring Revisited: The Role of the Comparative Question

**DOI:** 10.1371/journal.pone.0086056

**Published:** 2014-01-14

**Authors:** Ina Grau, Gerd Bohner

**Affiliations:** 1 Department of Social and Legal Psychology, University of Bonn, Bonn, Germany; 2 Department of Social Psychology, University of Bielefeld, Bielefeld, Germany; University of Leicester, United Kingdom

## Abstract

When people estimate a numeric value after judging whether it is larger or smaller than a high or low anchor value (comparative question), estimates are biased in the direction of the anchor. One explanation for this anchoring effect is that people selectively access knowledge consistent with the anchor value as part of a positive test strategy. Two studies (total N = 184) supported the alternative explanation that people access knowledge consistent with their own answer to the comparative question. Specifically, anchoring effects emerged when the answer to the comparative question was unexpected (lower than the low anchor or higher than the high anchor). For expected answers (lower than the high anchor or higher than the low anchor), however, anchoring effects were attenuated or reversed. The anchor value itself was almost never reported as an absolute estimate.

## Introduction

In a classic experiment, participants were asked whether the percentage of United Nations member states that are African is larger or smaller than a given number (the anchor value), which was ostensibly determined by spinning a wheel of fortune [1]. Later participants were asked to estimate the exact percentage of African UN member states. Regardless of the anchor values’ arbitrary nature, participants’ absolute estimates were assimilated to it: If the anchor was 10, participants’ mean estimate of the true value was 25, if the anchor was 65, their mean estimate was 45. Such assimilation of a numerical estimate toward a previously presented figure is called an anchoring effect. It was demonstrated in various domains, including knowledge questions [2]–[3], probability estimates [4], price estimates [5], sentencing decisions [6], and judgments about one’s own behavior [7]. The effect is stable over time [8] and independent of participants’ motivation [9] or expertise [10].

A common paradigm for studying anchoring effects uses a two-question sequence. First, the anchor is presented as part of a comparative question (e.g., “Is the Eiffel Tower higher or lower than X meters?”). A second question asks them for an absolute estimate (e.g., “What is the exact height of the Eiffel Tower?”). Typically, participants’ absolute estimate is shifted in the direction of the anchor value “X”.

In this article, we first discuss existing explanations for the anchoring effect [11]. We then argue that an important – but previously ignored – aspect of the questioning sequence is participants’ *answer* to the comparative question. In two studies, we empirically demonstrate that the answer to the absolute question depends on the answer to the comparative question.

### Conceptual Explanations

The anchoring effect was explained as a result of inadequate adjustment from a starting point: Participants start with the anchor value and adjust their estimate until a plausible value is reached; the adjustment is usually insufficient, resulting in a biased estimate [1]. A second explanation stresses rules of conversation [12]: Participants assimilate their estimate to an experimenter-provided anchor value because they expect the experimenter to be a cooperative communicator who presents plausible anchor values that are near to the correct answer. A third explanation is numeric priming: Large (vs. small) anchor values make large (vs. small) numbers more accessible in memory [13].

The fourth account, which is currently accepted as a sufficient explanation for the effects of anchors, is the selective accessibility model [3], [14]. The authors distinguish two processes: hypothesis-consistent testing and semantic priming. Accordingly, participants test the hypothesis that the critical value equals the anchor value, and generally prefer hypothesis-consistent testing [15]. If, for example, the comparative question reads, “Is the annual mean temperature in Germany higher or lower than 20°C?”, it may be obvious to test the hypothesis that the temperature is indeed 20°C, rather than any other hypothesis [3]. During hypothesis testing, participants are assumed to generate information that is compatible with the idea that the anchor value is correct. This information will thus be selectively more accessible and will be used for estimating the absolute value [16].

In addition to the anchor value itself, the selective accessibility model also considers the wording of the comparative question. Authors proposed that the comparative question “Is the river Elbe *longer* than 890 kilometers” would lead to a positive testing strategy in which participants generate knowledge consistent with the idea that the Elbe is indeed longer than 890 km. Conversely, the question “Is the river Elbe *shorter* than 890 kilometers” would lead participants to generate knowledge consistent with the idea that the Elbe is indeed shorter than 890 km [3]. The results supported this prediction, showing higher estimates in the “longer” conditions than in the “shorter” conditions, over and above the typical anchoring effect. In the case of non-directional comparative questions, however (e.g., “Is the river Elbe *longer* or *shorter* than 890 km”), authors assume that participants test the hypothesis that the target is *equal* to the anchor [3].

### A Neglected Variable: The Answer to the Comparative Question

Surprisingly, a variable that has received little attention in the anchoring paradigm is participants’ answer to the comparative question. Given that participants are asked to decide whether the target value is *smaller or larger* than the anchor value, it seems implausible that they test the hypothesis that the target value is exactly equal to the anchor. In fact, the only value that participants should *not* consider to be correct is the anchor value itself, because the comparative question “Is the value *smaller* or *larger* than X?” precludes the possibility that the value is exactly equal to “X”. Therefore, participants should rarely, if ever, report the anchor value as the correct answer to the absolute question. Instead, absolute estimates should be distributed around, but exclude, the anchor value. Participants should generate information that is compatible with their own answer to the comparative question.

Despite this plausible link between the answer to the comparative question and the answer to the absolute question, most studies on the anchoring phenomenon fail to report the distribution of answers to the comparative question, except of two studies [3], [17]. Although the link between the answers to both questions was not reported in either study, both reported the percentage of *unexpected* answers to the comparative question. Such are answers that estimate the true value to be even lower than the low anchor, or even higher than the high anchor. Interestingly, the percentage of unexpected answers was higher than would be expected based on the distribution of absolute estimates in pilot studies where no comparative questions had been asked. Authors explained this by assuming that an anchoring effect may already have occurred while participants answered the comparative question, and that a subsequent adjustment process would thus not be necessary for an anchoring effect on the absolute judgment to emerge [17]. In contrast, we argue that an adjustment process is necessary. Participants are informed by the wording of the comparative question that the anchor value must be wrong (although perhaps near to the correct value) and have to decide in which direction the correct value deviates from the anchor. Once they have decided this by answering the comparative question (“the correct value is lower/higher than the anchor”) the direction of adjustment is determined. Participants should generate further information that is consistent with their own answer to the comparative question instead of the anchor value itself. As a consequence, the answer to the absolute question will almost always be consistent with the answer to the comparative question (as was the case in a previous study) [17].

This means that anchoring effects on the absolute question should generally be more pronounced for people who give *unexpected* answers to the comparative question by assuming that the true value is even more extreme than the anchor value (i.e., higher than the high anchor or lower than the low anchor). By contrast, for people who give *expected* answers to the comparative question by assuming that the true value is less extreme than the anchor value (i.e., lower than the high anchor or higher than the low anchor), anchoring effects should be attenuated. If anchor values are relatively close to the true value, anchoring effects may even reverse for those people who give expected answers to the comparative question.

In two studies we used general knowledge items as the target estimates. To present anchor values in Study 1, we used nondirectional comparative questions. In Study 2, we used directional comparative questions of the type “Is the river Elbe *longer* than 693 kilometers?” [3]. In both studies, we first analyzed the consistency of answers to the absolute question; we predicted that most answers would be in line with the answer to the comparative question. Second, we predicted that an overall anchoring effect would emerge, with high anchors leading to higher absolute estimates than low anchors. Third, and most important, we predicted that the size and direction of the anchoring effect would be qualified by participants’ self-generated answers to the comparative question: A clear-cut anchoring effect would be obtained for participants who give unexpected answers to the comparative question; for participants who give expected answers to the comparative question, the anchoring effect would be attenuated or, in the case of less extreme anchor values, reversed. To test the latter prediction, we varied the distance of anchor values from the means of non-anchored pilot estimates.

## Study 1

### Method

#### Ethics statement

Procedures were approved by the Ethics Committee of the Faculty of Psychology and Sports Science at the University of Bielefeld. Following recommendations by the Ethics Committee, all participants provided oral informed consent, which was ensured and documented by the experimenter. We did not obtain written informed consent in order to protect participants anonymity.

#### Participants

Participants were 106 student volunteers from the University of Bielefeld (mean age = 23.95 years; SD = 8.74). The study’s alleged aim was to optimize the wording for general knowledge questions.

#### Materials

The questionnaire contained 25 knowledge items, including 6 from other authors [14]. Based on absolute estimates from a pilot study (N = 45; see Table 1), four questionnaire versions were produced. To vary the distance between anchor values at two levels, high and low anchor values were set at either 1 SD or 0.5 SD above and below the pilot study mean, respectively. Answers to the comparative question could thus be unexpected (lower than the low anchor or higher than the high anchor) or expected (higher than the low anchor or lower than the high anchor). Item content was counterbalanced with high versus low anchors: In one condition 12 items were paired with a low anchor and 13 with a high anchor; in the other condition this pairing was reversed.

**Table 1:** Pilot study (N = 45): Means and standard deviations of knowledge items.

	Item	Mean	Std. Dev.
1	Height of Mount Everest (m)	7222	2190
2	Length of a blue whale (m)	18	13
3	Length of the river Mississippi (km)	1186	871
4	Age of Mahatma Gandhi when he died	73	13
5	Number of bones in human body	180	82
6	Length of the river Elbe (km)	393	300
7	Albert Einstein’s first visit to USA (year)	1926	15
8	Elevation of city of Ulm above sea level (m)	235	205
9	Leonardo da Vinci’s year of birth	1636	175
10	Year of first Beatles LP	1962	4
11	Yearly beer consumption perhead in Germany (l)	89	68
12	Columbus in America (year)	1541	106
13	Age of Goethe when he died	60	15
14	Publication year of Freud’s“Interpretation of Dreams”	1905	25
15	Temperature inside a cigarette (°Celsius)	410	355
16	Height of Cheops Pyramid (m)	154	90
17	Number of inhabitants of Malta	259,525	226,317
18	Martin Luther’s theses (year)	1572	119
19	Melting point of iron (°Celsius)	812	423
20	Beethoven’s year of birth	1773	68
21	Men’s long jump world record (cm)	859	89
22	Weight of men’s discus (grams)	1802	1025
23	Vincent van Gogh’s year of birth	1781	114
24	Year of formation of the UNO	1952	10
25	Number of European Unionmember states since 2004	23	5

#### Design and procedure

Participants were randomly assigned to the conditions of a 2 (distances of the anchor values: low, high)×2 (content versions) experimental design. They completed the questionnaire in a laboratory with 1 to 5 people present at any one time. For all 25 items, participants first answered a comparative question (e.g., “Was the first Beatles LP published before or after 1964?”) by marking one of two response options (e.g., ‘before’ or ‘after’). Then they answered the respective absolute question (e.g., “In which year was the first Beatles LP published?”) by writing their response in an empty space. After completing the questionnaire, participants were debriefed and received a chocolate bar.

### Results

#### Preliminary analyses

We first analyzed the distribution of extreme answers to the comparative question in relation to the pilot results. Because anchor values were set at 1.0 SD or 0.5 SD below and above the mean value, expected percentages of extreme answers (“higher than the high anchor” or “lower than the low anchor”) – assuming a normal distribution – were 15.87 and 30.85, respectively. The percentages of extreme answers we observed in Study 1 were 27.12 in the 1.0 SD condition and 37.48 in the 0.5 SD condition. Thus, in line with previous findings [3], [17], we observed a higher percentage of extreme estimates than would be expected based on pilot data. This result suggests that an anchoring effect may indeed occur already when people answer the comparative question.

In a second step we analyzed answers to the absolute question in relation to the respective comparative question. We distinguished between consistent, inconsistent, and anchor estimates. For example, if a participant answered the comparative question “Was the first Beatles LP published before or after 1964?” by selecting “before,” any estimate below 1964 would be consistent, any estimate above 1964 would be inconsistent, and an estimate of exactly “1964” would be an anchor estimate. The number of estimates given was 2650 (106 participants×25 items). Of these, 2577 (97.25%) were consistent, 47 (1.77%) were inconsistent, 23 (0.87%) were missing values, and only 3 (0.11%) exactly equaled the anchor value.

#### Test of anchoring effects on the absolute estimates

Absolute estimates were averaged across items after being z-transformed based on item-wise distributions. To test our hypotheses, we computed four scores for each participant: the mean of all absolute estimates combined with low anchors in which the answer to the comparative question was as expected (i.e., higher than the low anchor), the mean of all absolute estimates combined with high anchors in which the answer to the comparative question was as expected (i.e., lower than the high anchor), and the analogous means for unexpected answers to the comparative question (i.e., where the response was lower than the low anchor and higher than the high anchor, respectively).

We conducted a 2 (anchor distance: large vs. small)×2 (low vs. high anchor)×2 (expected vs. unexpected answers) analysis of variance with repeated measures on the second and third factor. (The content factor – i.e., which items were paired with low or high anchor values – produced no effects and was thus dropped.) Means are shown in Table 2. Overall, the anchoring effect was significant (M_high anchor_ = .49 vs. M_low anchor_ = −.57), F (1, 104) = 1080.22, p<.001. Furthermore, the anchoring effect was larger when the distance between anchors was large (M_high anchor_ = .68 vs. M_low anchor_ = −.81) rather than small (M_high anchor_ = .33 vs. M_low anchor_ = −.36), as indicated by a significant interaction effect between anchor value and anchor distance, F (1, 104) = 142.64, p<.001.

**Table 2:** Estimates by Anchor Condition, Anchor Distance, and Answer to Comparative Question (Study 1).

	Answer to Comparative Question			
	Expected		Unexpected	
	Anchor Distance from Pretest Mean^a^			
Anchor Condition	0.5 *SD*	1 *SD*	0.5 *SD*	1 *SD*
High Anchor	−0.15	0.11	0.81	1.24
Low Anchor	0.11	−0.28	−0.83	−1.34
Difference: High minus Low	−0.26	0.39	1.64	2.58
t-Test for Difference^b^	−4.18	5.72	21.13	25.73
Cohen’s *d*	−0.94	1.29	4.04	5.39

^a^ Number of cases for the 0.5 *SD* and 1 *SD* anchor distance conditions was 56 and 50, respectively.

^b^ All t-tests were significant (*p*<.001).

Most importantly, the anchoring effect was qualified by the answer to the comparative question, F (1, 104) = 551.12, p<.001. Supporting our central hypothesis, the anchoring effect was large and in the expected direction only when the answer to the comparative question was unexpected (M_high anchor_ = 1.02 vs. M_low anchor_ = −1.07), t(105) = 26.91, p<.001. However, when the answer to the comparative question was in the expected direction, the anchoring effect was absent (M_high anchor_ = −.03 vs. M_low anchor_ = −.08), t(105) = 0.94. No other main or interaction effects reached significance. Nonetheless, simple effects of the anchor condition at each level of anchor distance by answer to the comparative question are displayed in Table 2.

### Discussion

Replicating earlier studies [14], we observed an anchoring effect, and this effect was larger when the distance between anchors was large rather than small. More importantly, we demonstrated for the first time that absolute estimates strongly depend on answers to the comparative question. Clear-cut positive anchoring effects were observed only for those participants who gave unexpected answers to the comparative question, judging the true answer to be lower than the low anchor, or to be higher than the high anchor. This pattern supports our assumption that people access information consistent with their own answer to the comparative question, rather than with the anchor value itself. When that answer was “lower” (“higher”), participants seemed to access information that was consistent with low (high) values. For participants who gave the expected answers to the comparative question, judging the true answer to be higher than the low anchor, or to be lower than the high anchor, the anchoring effect was attenuated or, when the distance between the two anchors was small, even reversed.

Furthermore, participants almost never reported the exact anchor value as their absolute estimate. If the answer to the comparative question was “lower” (“higher”), the absolute estimate was generally consistent with a lower (higher) value. This result is difficult to reconcile with the assumption of a positive test strategy in which participants test the hypothesis that the anchor value itself is correct. Instead, the result supports our hypothesis that participants’ own answer to the comparative question primed different informational contents, which in turn influenced their absolute estimates.

Finally, we fully replicated the result [17] that the amount of unexpected or extreme answers is greater than would be expected based on pilot estimates. These findings support authors conjecture that an anchoring effect may occur already when people answer the comparative question.

## Study 2

In our second study we tested the relevance of the answer to the comparative question for another assumption of the selective accessibility model. It was argued [3] that the wording of the comparative question may influence the test strategy people use and thus the kind of information that comes to mind. Accordingly, a question like “Is the river Elbe *longer* than 543 kilometers?” would facilitate a positive test strategy that leads participants to generate information consistent with a longer river. Conversely, the question “Is the river Elbe *shorter* than 543 kilometers?” would lead to the generation of information consistent with a shorter river. As a result, absolute estimates should be higher in the ‘longer’ condition than in the ‘shorter’ condition [3].

Replicating this design, we varied the directional wording of the comparative question. However, we again hypothesized that the *answer* given to the comparative question would strongly moderate the size of the anchoring effect, independent of any effect of question wording. For example, answering the question “Is the Elbe longer than 543 km?” with “no” should make similar information cognitively accessible as answering the question “Is the Elbe shorter than 543 km?” with “yes”, and should thus produce largely equivalent results. Furthermore, we analyzed the amount of consistent and inconsistent absolute estimates, as in Study 1.

### Method

#### Participants

Participants were 78 student volunteers from the University of Bielefeld (mean age = 22.71 years; SD = 5.92). The study’s alleged aim was to optimize the wording of general knowledge questions.

#### Materials

We used the same 25 items as in Study 1, and item content was again counterbalanced with anchor condition. High (low) anchor values were set to either 0.5 SD or 1.0 SD above (below) the pilot mean. A new independent variable was the wording of the comparative question, which was varied such that an affirmative answer would imply a value that was either higher or lower than the anchor. For example, in the high anchor condition the item about the river Elbe read either “Is the Elbe shorter than 543 kilometers?” or “Is the Elbe longer than 543 kilometers?” (response alternatives: “yes” and “no”). Absolute questions were the same as in Study 2, for example “How long is the Elbe?,” with an open space in which participants could write their answer.

#### Procedure

The procedure was the same as in Study 1. Participants were randomly assigned to the conditions of the 2 (content versions)×2 (anchor distance: low vs., high)×2 (wording: higher vs. lower) design.

### Results

#### Preliminary analyses

Expected percentages of extreme answers to the comparative question were again 15.87 and 30.85, respectively, for the 1.0 SD and 0.5 SD conditions. The observed percentages, however, were 33.25 in the 1.0 SD condition and 40.05 in the 0.5 SD condition. Again, an anchoring effect thus seemed to occur already when participants answered the comparative question [17].

Authors of the selective accessibility model predicted a positive test strategy whereby participants test the hypothesis that the information given in the wording of the comparative question (e.g., “Is the Elbe *shorter* than 543 kilometers?”) is correct [3]. This would lead to more “yes” answers than “no” answers. In the present study, the percentage of “yes” answers was 48.82, whereas the percentage of “no” answers was 49.96 (1.49% missing values). A tendency to say “yes” was thus not found.

In the next step we analyzed the distribution of absolute estimates in relation to answers to the respective comparative question. Absolute estimates could again be consistent or inconsistent. If, for example, one says the Elbe is longer than 543 km (“yes”), any estimate above 543 is consistent with the answer to the comparative question. Among the 1950 absolute estimates, we found 1746 (89.54%) to be consistent and 96 (4.92%) to be inconsistent; answers were missing 44 times (2.26%), and the exact anchor value was estimated 64 times (3.28%). This latter percentage was higher than that observed in Study 1, which may be due to the fact that answering the absolute question with the exact anchor value is not in conflict with the answer “no” in the comparative question. If one answers the question “Is the Elbe longer than 543 km” with “no”, the Elbe can be either shorter than 543 km or exactly 543 km long. Estimating the exact anchor value thus contradicts the answer to the comparative question only if that answer was “yes”. Such conflicting estimates were given only 5 times (0.26%).

#### Test of anchoring effects on the absolute estimates

As in Study 1, composite indices across items were computed after z-transforming all absolute estimates. For each participant, four values were computed: the mean of all answers to questions combined with low anchors in which the answer to the comparative question was as expected (“value is higher than low anchor”), the mean of all answers to questions combined with high anchors in which the answer to the comparative question was as expected (“value is lower than high anchor”), and the analogous means for unexpected answers to the comparative question (“value is lower than the low anchor”; “value is higher than the high anchor”).

Counterbalancing of item content again produced no effects, so this factor was dropped. For analyzing absolute estimates, we conducted a 2 (anchor distance: large vs. small)×2 (wording: lower vs. higher)×2 (low vs. high anchor)×2 (expected vs. unexpected answers) mixed analysis of variance with repeated measures on the third and fourth factor. Means are shown in Table 3. The anchoring main effect was replicated (M_high anchor_ = .45 vs. M_low anchor_ = −.52), F (1, 74) = 752.37, p<.001. Again, the anchoring effect was larger when the anchor distance was large (M_high anchor_ = .61 vs. M_low anchor_ = −.70) rather than small (M_high anchor_ = .28 vs. M_low anchor_ = −.33), as indicated by a significant interaction effect of anchor condition and anchor distance, F(1, 74) = 103.28, p<.001.

**Table 3:** Estimates by wording, anchor condition, and answer to comparative question (Study 2).

	Answer to Comparative Question							
	Expected Answers				Unexpected Answers			
	Anchor Distance from Pretest Mean^a^							
	0.5 *SD*		1 *SD*		0.5 *SD*		1 *SD*	
	Wording of the Comparative Question							
Anchor Condition	Lower	Higher	Lower	Higher	Lower	Higher	Lower	Higher
High Anchor	−.18	−.23	.10	.33	.74	.78	.98	1.06
Low Anchor	.02	.08	−.39	−.35	−.72	−.68	−1.08	−.99
Difference: High minusLow	−.20	−.31	.49	.68	1.46	1.46	2.06	2.05
t-test for Difference^b^	−1.74+	−2.61*	4.44***	7.24***	8.64***	15.13***	21.59***	13.87***
Cohen’s *d*	−0.62	−1.09	0.69	2.58	4.71	2.78	4.82	5.23

^a^ Number of cases was 21 in the wording “lower” condition, and 17 in the wording “higher” condition in the 0.5 *SD* condition and 20/20 in the 1 *SD* condition.

^b^+ *p*<.10;

* *p*<.05;

*** *p*<.001.

Most importantly, we again obtained an interaction effect of anchor condition and answer to the comparative question, F (1, 74) = 247.05, p<.001. As predicted, the anchoring effect was larger when answers to the comparative questions were unexpected (M_high anchor_ = .89 vs. M_low anchor_ = −.87), t (77) = 23.76, p<.001, than when they were expected (M_high anchor_ = .01 vs. M_low anchor_ = −.17), t (77) = 2.45, p<.05. In case of a low anchor distance and expected answers to the comparative question, the anchor effect was again reversed (see Table 3 for simple effects of anchor condition within combinations of the other factors). No main or interaction effect of wording was found, all F<1.

### Discussion

In contrast to the argumentation and findings of [3], we found no evidence for a test strategy effect in the direction suggested by the comparative question. Instead, answers of “yes” and “no” to that question were equally frequent. The wording of the comparative question (“Is the Elbe *longer* vs. *shorter* than 543 km?”) in itself also did not affect absolute estimates. Instead, the overall anchoring effect, which was again replicated, was once more strongly qualified by answers to the comparative question. In the case of low anchor distances, an anchoring effect occurred only when the answer to the comparative question was unexpected, whereas it reversed when that answer was expected, replicating the pattern found in Study 1.

Extreme answers to the comparative questions again occurred more often than would be expected by chance. An anchoring effect thus seemed to be present already in answers to the comparative question [17].

## General Discussion

If people are asked to estimate a numerical value and do not know the correct answer, they are influenced by an anchor value that is presented before in a comparative question. The most elaborate explanation for this effect is the selective accessibility model [3], [14], which holds that participants first try to verify the hypothesis that the anchor is correct when answering the comparative question, and then, in a second step, use anchor-consistent information to answer the absolute question.

Taking the selective accessibility model as a starting point, we presented the hypothesis that people’s answers to the comparative question have a decisive impact on their subsequent cognitions. In most studies to date participants’ answers to the comparative question had been completely ignored. But even when the distribution of answers to the comparative question was reported, these answers were not included as a predictor of answers to the absolute question [3]. If a person decides that the exact value is smaller than a low anchor (or larger than a high anchor), only values that are more extreme than the anchor are likely to be considered as possible results of the absolute estimate. The search for information is thus restricted to content that is compatible with such extreme values, and will thus contribute to a large anchoring effect. The situation is different for a person who, more correctly or expectedly, decides that the true value is larger than a low anchor or smaller than a high anchor. Now, only values that are less extreme than the anchor are likely to be considered as possible results of the absolute estimate. The search for information is restricted to content that is compatible with such less extreme values, and will thus contribute to an attenuated anchoring effect, or even a reversal of the anchoring effect if anchor distances are small.

In two studies, we found unequivocal support for this new hypothesis: Anchoring effects generally replicated, but their magnitude and even direction were qualified by answers to the comparative question. These results can specify one of the explanations [1] that participants start at the anchor value and adjust insufficiently. More precisely, some participants decide to adjust in the wrong direction and others in the correct one. If one only considers the mean values for high and low anchor conditions, insufficient adjustment appears to occur. If, however, one considers the means separated in expected and unexpected answers to the comparative question, anchor effects are absent following the expected answer to the comparative question. The “insufficiency” is thus not due to the amount of adjustment but due some people’s starting out in the wrong direction.

In contrast to the assumption of the selective accessibility model that participants positively test the hypothesis that the anchor value is correct, the anchor value was almost never reported as the true value in absolute estimates. Instead, participants seem to test the hypothesis that their own answer to the comparative question is correct. If one gives the answer that a value is larger (smaller) than a given anchor, search of further information is restricted to values that are larger (smaller) than the anchor. Nonetheless, the results are in line with the assumption that participants believe that the anchor value is not totally wrong and that the correct value has to be near the anchor. This restatement would be compatible with the selective accessibility model and with the explanation that participants expect the experimenter to present plausible information [12].

The effect of the answer to the comparative question was independent of that question’s directional wording. According to the selective accessibility model, the variation in wording used in Study 2 should have induced different test strategies in line with its direction (“is the value lower than X” versus “is the value higher than X”). However, we did not observe any effect of question wording on the absolute estimates, but again a strong moderating effect of participants’ *answer* to the comparative question, regardless of its wording. Taken together, our findings imply that the wording of the comparative question is influential only in combination with the *answer* to that question. If one says “yes” to the question of whether a value is larger than x, further considerations are restricted to larger values. If one says “no,” the true value can be either lower than or equal to the anchor value. The answer to the absolute question always seems to be a consequence of the answer to the comparative question.

These observations shift our focus to the question of what determines the answer to the comparative question itself. Our analysis of the distribution of unexpected answers showed that unexpected answers were more frequent than would be expected by chance. These findings support a conjecture [17] that anchoring may occur already at the stage where participants answer the comparative question. However, our reasoning is not in line with their assumption that a subsequent adjustment process toward the true value takes place. Instead we assume that the wording of the comparative question forces participants to adjust their absolute estimates away from the anchor in the direction (higher or lower) they decided while answering the comparative question.

Our findings may have implications for judgments in natural contexts, given the pervasive importance of social comparisons [18]. Many everyday situations resemble the current paradigm in that people often ponder (and answer) a comparative question before coming up with an exact estimate of a numeric value. For example, a judge or jury may first consider whether the prosecutor’s demand is too high or too low, and only later determine a sentence. Research should thus routinely consider the role of *answers* to initial, anchor-related relative questions as a possible co-determinant of later judgments.
